# Animal models in the study and treatment of orofacial pain

**DOI:** 10.4317/jced.55429

**Published:** 2019-04-01

**Authors:** Miguel-Ángel Martínez-García, Blanca C. Migueláñez-Medrán, Carlos Goicoechea

**Affiliations:** 1 PhD, Visiting Professor. Area of Pharmacology, Nutrition and Bromatology. Department of Basic Health Sciences. School of Health Sciences. Universidad Rey Juan Carlos (URJC), Alcorcón, Madrid (Spain) – I+D+i Medicinal Chemistry Institute (IQM) associated unit, (CSIC); 2 DDS, PhD. Adjunct Professor. Area of Stomatology. Department of Medicine and Surgery, Psychology, Preventive Medicine and Public Health, Immunology and Medical Microbiology, Nursing and Stomatology. School of Health Sciences. Universidad Rey Juan Carlos (URJC), Alcorcón, Madrid, Spain; 3 PhD, Professor. Area of Pharmacology, Nutrition and Bromatology. Department of Basic Health Sciences. School of Health Sciences. Universidad Rey Juan Carlos (URJC), Alcorcón, Madrid (Spain) – I+D+i Medicinal Chemistry Institute (IQM) associated unit, (CSIC)

## Abstract

**Background:**

Pain is one of the first causes of medical consultation in the world and by extension of dental consultation too. Orofacial pain comprehends the oral and facial regions including teeth, oral mucosa, gingiva, tongue and lips, but also the muscles of the jaw and neck, the temporomandibular joint, face, head and neck. Despite its highly estimated prevalence, it appears controversial and hard to quantify given the lack of common criteria to select the population under study and the difficulties to classify the different types of pain. Although for many patients the problem eventually fades after tissue healing, certain sub-chronic and chronic pain conditions remain notoriously undertreated. In this respect, animal models can be of great help.

**Material and Methods:**

A systematic search was conducted in PubMed-Medline with appropriate keywords: orofacial pain, prevalence and dentist. Seven groups were generated and a second search based on each of these groups and on animal models was made. Search was restricted to English and Spanish, but no time restriction was applied.

**Results:**

There are as yet few experimental models of orofacial pain: there hardly exists no other than trigeminal nerve injury for neuropathic pain, a bunch of oral squamous cell carcinoma models (mainly referred to the tongue) for cancer pain and none for the painful swelling of salivary glands. Similarly occurs for the burning mouth syndrome. A few more exist for inflammatory odontalgiae, aphthae, joint, myofascial and muscle inflammatory pains, although scarcely diverse as regards the nature of the noxious stimulus.

**Conclusions:**

Given the relevance of envisaging the mechanistic of the various types of orofacial pain, new experimental models are needed on the basis of the dentist’s perspective for their correct management.

** Key words:**Orofacial pain, neuralgia, odontalgia, oral cancer, animal models.

## Introduction

Pain is one of the most frequent reasons for consultation in primary health care in the world (1). Although a highly variable prevalence has been estimated in the global population (~10-55% as stated by the International Association for the Study of Pain, IASP), today a prevalence of 19% is generally accepted for chronic pain in Europe (2). The elaboration of statistics appears, however, to be a challenging task for diverse reasons: the varied origin of the sample, the sex and age ratios of the sample population, the duration of the ailment, the data collection strategy or an overly lax nomenclature for different mechanisms and types of orofacial pain. Moreover, cultural and socioeconomic conditions may also influence (3-6).

Orofacial pain is often referred, thereby interfering with its diagnosis. However, according to the IASP, for a significant number of patients pain may persist after the pain-producing stimulus is removed or even in the absence of a clear physical cause or illness. Such pain is considered chronic and generally requires a multidisciplinary and integrated approach.

Although the main, and often only, transmission pathway considered is the trigeminal nerve, the fifth (trigeminus), seventh (facialis) and ninth (glossopharyngeus) cranial nerves also contribute for most of the orofacial pain. Therefore it is not surprising that orofacial chronic pain is so difficult to classify and to cope with, especially in regard to some atypical algiae. In this respect, animal models can prove a good tool to understand the mechanisms and management of orofacial pain.

## Material and Methods

Firstly, a PubMed-Medline search was made using the following research sequence: “orofacial pain” AND “prevalence” AND “dentist” (n = 145 articles). In selecting the studies, we reviewed the titles and abstracts to identify relevant publications, of which the complete text was then obtained. Search was restricted to English and Spanish, but no time restriction was applied. Based on these preliminary results, seven groups were generated: odontalgia (dental), oral mucositis (oral ulcer or inflammatory), muscle (or masseter), temporomandibular, neuropathic (trigeminal or facial), burning mouth syndrome and oral cancer (tongue or facial cancer). Then a second search was made with the terms: “animal model” (alternatively rat or mouse) AND “orofacial pain” AND each of the seven groups just cited. Same restriction criteria as above were used.

## Results

The most common orofacial disorders that can evolve with pain are odontalgia, soft tissue mucosa ulceration, muscle and joint pain and neuralgiae; considering burning mouth syndrome and cancer pain as groups apart.

-Animal models of odontalgia.

**Figure 1:**
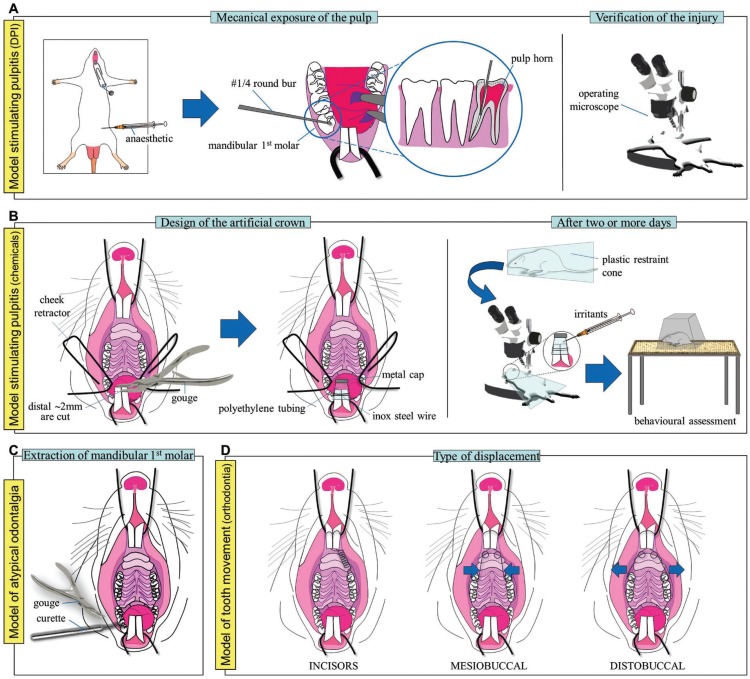
A) Model stimulating pulpitis: dental pulp injury (DPI) model. B) Model stimulating pulpitis: application of chemical irritants to incisors. C) Model of atypical odontalgia. D| Model of tooth movement (orthodontia). Refer to text for a more detailed explanation.

One of the first dental inflammatory pain models developed in rodents consists in the mechanical exposure of the pulp of two alternate or the three mandibular and/or maxillary molars with a round bur (#2) at high speed in rats (7). Although pulpitis and periapical lesions correlate with the ailment observed in patients, the reproducibility of the model is questionable: different dental crowns may exhibit varying degrees of damage. And hence, pulpal and neural pooled responses might be rather inaccurate, misleading or inconsistent. Today, mechanical exposure of both pulp horns of the mandibular (or maxillary) first molar in mice (or rats) is more commonly used (8) (Fig. 1A). Unlike the previous case, erosion of the occlusal surface of the tooth may be performed uni- or bilaterally by means of a dental drill with round bur (#1/4) at low speed and the injury extension visualised under an operating microscope to check that both pulp horns are affected. Body weight and water and food consumption can be monitored as proxy measures of general discomfort. In addition, the use of an infrared beam-based activity meter during daytime –rats are nocturnal– can be used to determine any possible alteration of the spontaneous locomotor activity at typically sleeping or rather resting moments. Increased frequency and duration of face rubbing on the fore- or hindlimbs towards the injured area and conditioned-reinforcement tests can also shed light on the spontaneous pain an animal is experiencing. The consumption of a sweet solution (water + sucrose), for instance, has been reported to be directly proportional to the magnitude of the nociceptive component that a mouse with dental pulp injury is suffering (9). That is, animals experiencing odontalgia drink more sweet solution than control animals. Further identification of anxiety behaviours by the open field or elevated plus maze tests will give a multidimensional insight of the pain being experienced by these animals (9).

Given that dentalgia also depends on the type of tooth and its innervation, an alternative to the previous models consists in the injection of proinflammatory agents in the rat mandibular incisor pulp (10) (Fig. 1B). Briefly, the distal 2 to 2.5mm of the mandibular incisors are carefully cut with a fine bone gouge forceps (rongeur) and an artificial crown of polyethylene tubing is placed so it wraps both incisors. A 1–2 mm sealed metal cap tops off the surface of the crown to close the incisal edge. Eventually, the opposite end of the crown is fixed on the incisors by a 0.01inch orthodontic stainless steel wire and rats are allowed to recover in their cages for at least two days before behavioural assessment; then, restrained in a plastic cone and a 10μl volume of the irritant substance is carefully administered into the artificial crown under an operating microscope. Capsaicin (10-100μg/μl; 10μl) or alternatively formalin (2.5%; 10μl) can be used, and behaviour is assessed immediately after a single injection of the irritant substance according to the following grading score system:

-score type of behaviour

 0 normal

 1 abnormal head movements (mild head shaking or placement of the jaw on the floor)

 2 continuous shaking of the lower jaw

 3 excessive rubbing of the mouth with the forelimbs 

The total time spent in each of the four scales is estimated over successive 3min blocks during a total time of 60min. Eventually, comparisons between the time-response curves in control and treated animals are made.

The extraction of mandibular first molars in rats could constitute a model to study peripheral and central sensitisation occurring in some atypical odontalgia (11) (Fig. 1C). However, its use remains merely residual so far.

Interestingly, some works on orthodontics have also been developed as models of tooth movement in rat. The orthodontic device commonly consists of a stainless steel coil spring attached to both upper incisors by a metal wire on one edge and to the maxillary first molar on the other –uni- or bilaterally (12)–. Optimum force magnitude and carrying duration varies greatly between the different works: from two days up to four weeks and from 10 to 100g. The orthodontic treatment eventually results in the displacement of teeth, affecting either the incisors or the oral cavity (mesio- or distobuccally) as required (Fig. 1D). Again, body weight and food consumption can be considered as indirect measures of general discomfort; altered sleep/wake patterns and anxiogenic behaviour as well.

-Animal models of oral mucositis-induced pain.

Oral ulcerations are relative common recurrent mucosal lesions often identified with pain; animal models for oral mucositis are most currently based on chemical lesions.

**Figure 2:**
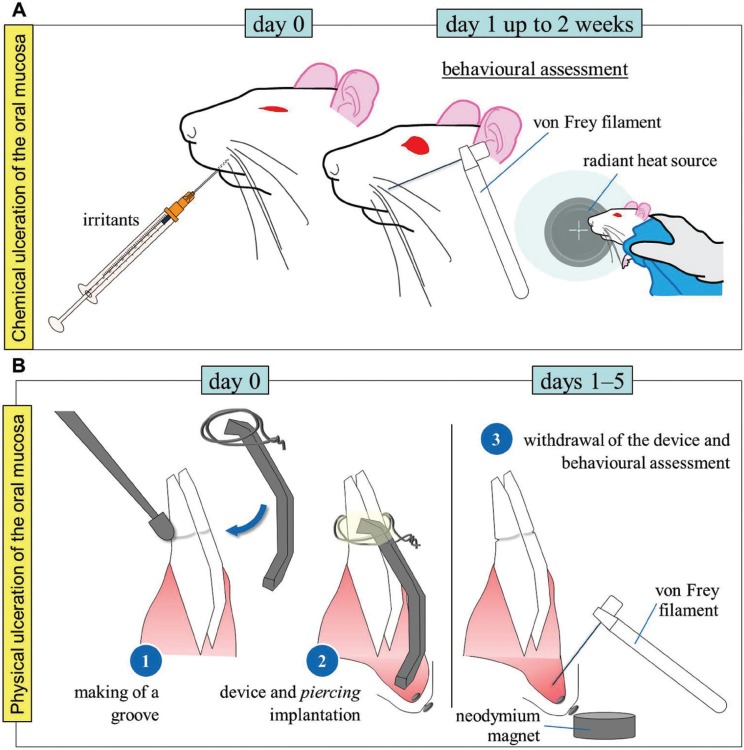
A) Model of chemical ulceration of the oral mucosa. Hydrochloric acid (1%; 20µl) or acetic acid (99.7%; 50µl) are injected into one side buccal mucosa. Evoked-pain responses to light touch and noxious heat are regularly assessed during the next 14 days. B) Model of physical ulceration of the oral mucosa. Piercing installation and ulcerative device implantation and removal are performed under anaesthesia. Upon device removal behavioural response is determined. Behavioural assessments (rubbing of the face or von Frey test) are always conducted at a fixed time on a daily basis during 5 days.

A model of pain induced by unilateral chemical ulceration of the oral mucosa consists in the injection of hydrochloric acid (1%; 20µl) into oral submucosa of athymic nude mice (13). The ulcer reaches its maximal extension the first day following chemical administration and gradually reduces during the next two weeks. As previously mentioned, regular assessment of body weight and food and water consumption may yield information on the level of discomfort experienced by the animal. For evoked pain, applying von Frey filaments or a radiant heat source to the external area of the cheek or vibrissae area will enable to assess the existence of tactile allodynia or heat hyperalgesia respectively (Fig. 2A).

Recently, a complex model of physical ulceration of the labial fornix of the oral mucosa consisting in a metal wire attached to the mandibular incisors of a rat has been developed (14). After one day, the device is withdrawn and the behaviour assessed during the next 5 days. The time spent rubbing the face with both forelimbs can be assessed during 10min daily to serve as a spontaneous pain measurement. Additionally, the existence of tactile allodynia can also be evaluated. For this purpose, previously to the implantation of the physical ulcerative device, a small piercing is installed just beneath the skin below the lower lip and above the chin in anaesthetised rats. Rats are then trained for 3-4 days to introduce their head (actually their perioral region) into a plastic or acrylic restrainer. After training fulfilment the device is implanted and, the following day, after removal, the use of a neodymium magnet when the rat enters the restrainer will enable to expose the ulcerative region to the researcher. The evoked-pain response (head withdrawal threshold) to von Frey hair stimulation onto the labial fornix will be determined (Fig. 2B).

-Animal models of inflammatory pain in the facial region.

The model of formalin-induced facial pain (15) consists in the administration of 2.5% formalin (50μl) into the facial vibrissae (one side upper lip lateral to the nose) of non-anaesthetised rats. Nociceptive behaviour is determined immediately after the injection. The time rats spend rubbing the injected area is calculated in blocks of 3min during a total time of 30min. A typical biphasic nociceptive response may be observed after formalin injection, with the first phase, between 0 and 3 min, reflecting the direct chemical stimulation of the nociceptive terminals, and the second phase, between 12 and 30 min, representing the orofacial inflammatory pain. The model of capsaicin-induced facial pain (2μg/50μl) (16) constitutes a modification of this model, and same behaviour is analysed.

Unlike the previous two models, the model of carrageenan-induced facial pain (100μg/50μl) (16) focuses on an evoked-pain response rather than on a spontaneous pain response. The latency to display head withdrawal or alternatively vigorous flicking of the snout upon stimulation of the vibrissae pad with a radiant heat source is registered before (baseline), immediately after carrageenan injection and every hour during the next 6h. A 20s cut-off time is used in order to prevent tissue damage. The model of Complete Freund’s Adjuvant (CFA)-induced pain (50μl) (17) in the vibrissae area of anaesthetised rats represents another model of facial inflammatory pain. Similar to the previous case, an evoked-pain response is registered: either the head withdrawal threshold or face stroking with the forepaw to von Frey hair stimulation of the vibrissae area.

-Animal models of muscle pain.

Myofascial pain is together with neuralgia one of the most complex types of orofacial chronic pain to treat and also possesses one of the highest incidence rates. Most experimental animal models, however, refer strictly to the masseter muscle: model of pain induced by the injection of glutamate (1–2M; 40μl) (18), model of pain induced by the injection of hypertonic saline (5% NaCl; 100μl) (19) or model of pain induced by the injection of CFA (15μl) (20) into the masseter muscle. All of them are based, as well, on the unilateral single administration of pro-inflammatory chemicals through a carefully inserted catheter. In the first case, glutamate is administered under isoflurane light anaesthesia and nociceptive behaviour is evaluated by applying von Frey filaments onto the skin surface of the injected area. Registers of the head withdrawal thresholds are noted 5, 15, 30, 45, 60, and 90min after injection on conscious (awake) animals. In the second case, both administration of hypertonic saline (HS) and behavioural assessment take place under light anaesthesia. HS produces a short acting and non-sensitising ipsilateral hindpaw shaking behaviour. In the third case CFA administration is injected under light isoflurane anaesthesia so the animals can rapidly recuperate. After 10min, animals are video-recorded for a 60-min period and the grooming patterns are further calculated in 10 blocks of 6min, considering the duration of time they spent grooming the injected area and the time that they occur. A fourth model of inflammatory pain in the masseter muscle consists in the injection of carrageenan (3-9%; 100μl) (21). A single administration of carrageenan is injected under no anaesthesia and nociceptive behaviour is evaluated by applying von Frey filaments, a pressure application measurement (PAM) device or a hot pen (45ºC) onto the skin surface of the injected area 4h, 1, 2, 7 and 14 days after injection. A 20s cut-off time is used for the latter test in order to prevent tissue damage.

Finally, a model of pain in the masticatory muscle of the temporal region induced by psychological stress has been developed (22). A communication box made of 16 compartments (dimensions: 16×16cm each) separated from each other by transparent plastic walls with numerous holes and a floor formed by a metal wire mesh is connected to a 48V generator. A plastic surface on top of the mesh is placed only in 8 alternate compartments. This way, although 16 rats can be introduced into the communication box, 8 of them will receive no electric discharge. However, these animals will be exposed to the screaming, urine and faeces odour and bounds of the neighbouring rats through the holes on the walls. During a first phase of 7 days, all rats are placed daily in the communication box for 1hour in order to accustom to the test conditions (in absence of any electric discharge). In a second phase of 14 days, the rats in direct contact with the metal wire will receive daily electric discharges every 2 seconds during 1 hour. An independent group of rats will be placed in a different communication box receiving no electric discharges during the 28-day time period as a control. The elevated plus maze test may be used (once per week) to assess the anxiety level in the animals. The von Frey test may be applied on the masseter and temporary muscles to evaluate the head withdrawal threshold –or alternatively the vocalization– to a tactile stimulus. Body weight can also be assessed as an indirect method to determine general discomfort all along the process. Rats located next to those receiving electric discharges will exhibit increased aversion to open spaces (anxiety symptom) in the elevated plus maze test and lower head withdrawal thresholds in the von Frey test than control rats (communication box not electrically stimulated) (22).

-Animal models of joint pain.

The administration of different substances into the temporomandibular joint (TMJ) accurately recreates the type of pain experienced by many patients with TMJ disorders: model of inflammatory pain induced by formalin injection (2.5%; 50μl) (15), model of inflammatory pain induced by CFA injection (50μl) (23) or model of inflammatory pain induced by injection of mustard oil (20%; 25μl) (24) into the TMJ. Besides inflammatory pain, mixed pain (that further combines nociceptive and neuropathic pain) has also been studied. In this respect, a model of osteoarthritis pain induced by monosodic iodoacetate (MIA; 0.5mg; 50μl) injection into the superior compartment of the TMJ has recently been developed (25). MIA injection affects the temporal fossa, but especially the condylar cartilage and the subchondral bone. Chondrocyte death already occurs within the first day, with a maximum pain peak after three days. The model was first aimed at the knee diarthrosis and is consistent with a biphasic nociceptive nature: an early inflammatory phase in the first week and a second, chronic phase, starting two weeks post-MIA injection (26).

In all the cited models, the existence of allodynia can be assessed as previously mentioned, by means of von Frey filaments. Body weight and water and food consumption can be used as indirect methods to quantify general discomfort, which, in combination with the open-field, hole-board, elevated plus maze and actimeter tests may provide information of the psychological aspect of pain.

-Animal models of neuralgia.

**Figure 3:**
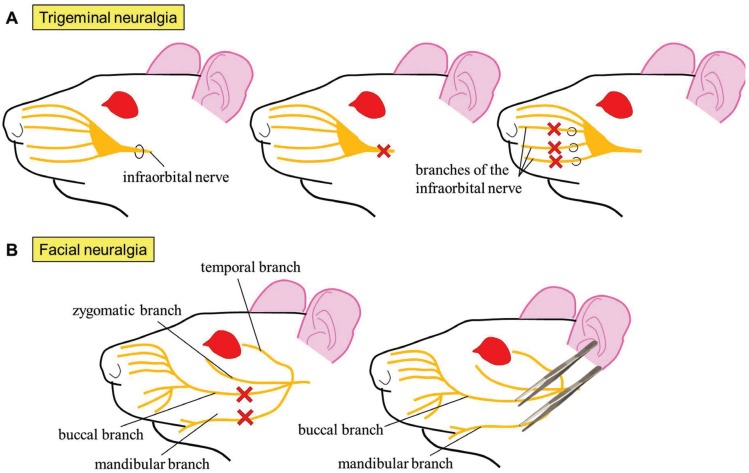
A) Animal models of trigeminal neuralgia. Ligation (upper left), complete transection (upper middle) or ligation with ulterior excision (upper right) of the infraorbital nerve or associated branches. B) Animal models of facial neuralgia. Complete transection (lower left) or clamping (lower right) of the buccal and mandibular nerves.

Orofacial neuropathic pain may result in a variety of lesions or disorders and nowadays represents a not easily solvable problem. Consequentially, numerous animal models have been developed: model of chronic constriction injury induced by ligation of the infraorbital portion of the trigeminal nerve (maxillary division) (27), model of pain induced by trigeminal selective transections or the spared nerve injury model induced by ligation and consequent excision of the main branches of the infraorbital nerve innervating the vibrissae (28). In all the three models, tactile evoked allodynia is assessed by means of von Frey filaments (29), although cold allodynia and heat hyperalgesia may additionally be evaluated (30) (Fig. 3A).

In the last years, a model of facial neuralgia has emerged (31). The model presents two variants. In the first one, the lesion is irreversible: approximately 1mm of the mandibular and buccal branches of the facial nerve are excised. In the second, the lesion is reversible: the branches aforementioned are compressed in one direction with a clamp for 30s and then perpendicular to this sense for other extra 30s (Fig. 3B). Although the model has only been used to assess the locomotor activity so far, microglial activation has been observed in primary motor cortex of vibrissae after both types of injury (31), indicating the existence of central sensitisation.

-Animal models of burning mouth syndrome.

The development and implementation of animal models of burning mouth syndrome constitutes still nowadays a challenge for basic research. Nevertheless, a model of zinc deficiency in rats (32) on a low-zinc diet (2.3 mg/kg) alternating weekly with a normal diet (50 mg/kg) in 20 weeks has been developed. Just the histology of the tongue has been analysed so far. However, food intake has been used as a symptom of general discomfort.

-Animal models of cancer pain.

Oral Squamous Cell Carcinoma (OSCC) is one of the most common malignant neoplasms and the most common neoplasm in the oral cavity (33). Despite improvements in the treatment of OSCC, high recurrence rates and morbidity make the use of animal models a good tool to help throw light on how to face cancer pain. More particularly when the mechanisms responsible for oral cancer pain are yet to be fully determined (34).

The model of tongue OSCC induced by 4-nitroquinoline 1-oxide (4-NQO) accurately reproduces the pathology occurring in humans: development of an OSCC accompanied by dysplasia, papilloma and tongue lesions. Hence, this model represents one of the most widely used methods to study oral cancer in both rats (35) and mice (36). The carcinogen 4-NQO is daily administered in the drinking water or alternatively brushed on the tongue of anaesthetised animals three times weekly during 16 weeks (37). The model of tongue OSCC induced by the implantation of the human HSC-2 cell line in athymic mice (38) constitutes a modification of this model. Decreased eating behaviours and food intake, generally associated to the psychological aspect of pain, have been identified in both models.

Carcinoma-induced pain has also been studied in other intra- and extraoral regions. The model of subperiosteal abscess-induced cancer pain consists in the subperiosteal implantation of the rat SCC-158 line into the lower gum in rats (39). Assessment of pain-evoked responses is performed as previously mentioned: on the one hand, mechanical sensitivity of the whisker pad (maxillary nerve region) and the submandibular skin (mandibular nerve region) is evaluated with von Frey filaments and, on the other hand, thermal sensitivity is determined by gently pressing the snout of the animal against a heat plate (55ºC). Head withdrawal thresholds and head withdrawal latencies (or alternatively the latency to vocalization) are registered respectively. Additionally, the model of OSCC induced by subcutaneous implantation of the rat Walker 256 cell line into the whisker pad in rats (34) reproduces in a precise way the typical spontaneous pain, allodynia and hyperalgesia developed in many patients with an extraoral facial cancer. The time that the animal spends performing face wash strokes directed to the injected area is registered during 10min at a fixed time on a daily basis during several days. Head withdrawal thresholds to von Frey hairs and head withdrawal latencies to a radiant heat source are also evaluated.

## Discussion

Orofacial pain and particularly oral pain have been underresearched and underrecognized for many years, compared to other types of pain in different anatomical regions. Furthermore, an overly lax nomenclature for the different mechanisms and types of orofacial pain has led to a lack of common criteria and to the existence of distinct nominations for a same type of pain. Moreover, the development of Orofacial Pain Units gathering together experts on the stomatognathic and nociceptive systems are still lacking.

In addition, although the clinical ethics committees for biomedical research consider the use of experimental animals a moral obligation prior to clinical assays, there is a shortage and scarce knowledge of experimental animal models for orofacial pain. The summary table below ( Table 1,  Table 1 continue) shows most of the different animal models existing for the distinct types of orofacial pain raised in this work.

**Table 1:**
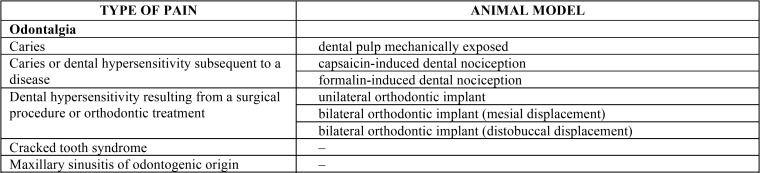
Summary table of the different animal models for the study of the numerous types of orofacial pain.

**Table 1 continue:**
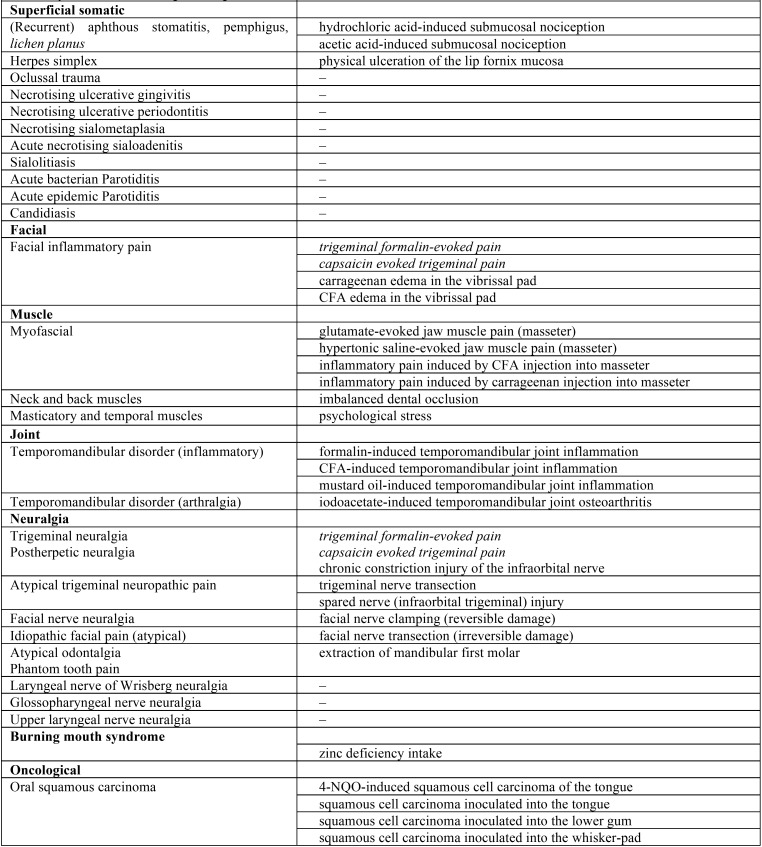
Summary table of the different animal models for the study of the numerous types of orofacial pain.
